# Open-Air Treatment of Phthisis as Practiced in German Sanitaria

**Published:** 1899-02

**Authors:** 


### OPEN-AIR TREATMENT OF PHTHISIS AS PRACTICED IN GERMAN SANITARIA.

Impure or "rebreathed" air is the principal predisposing cause of phthisis; the leading principle of the treatment, described by Dr C. T. Williams (*British Medical Journal and American Medico-Surgical Bulletin*), is that the consumptive should pass the greater part of his time in the open air, protected from weather, and as a rule in the prone position, and that at night he should sleep with windows open. This treatment is carried on either in covered balconies or terraces with one side open or in pavilions, some of which are arranged to rotate, the invalid being thus enabled to be always protected from wind and exposed to sunshine. The covered terraces are of considerable depth and height; the drifting rain and snow are kept out by curtains, and the too intense sunheat by blinds. The patient lies out from seven to eleven hours a day, only moving for meals and exercise, and going indoors at night. Thus protected, all kinds of weather are well borne, provided only the patients are well covered up in furs and wraps.

To resist cold the prone position is far better than sitting, probably because of the equalizing effect it has on the circulation of the body and the less strain it exerts on the heart. The author has found patients lying in comfort in the open air at a temperature of 4° F.; on feeling their hands and feet he found them perfectly warm. Thus the patient is hardened against fresh cold, his appetite is increased, his sleep is promoted, his night sweats reduced, and his pyrexia is lowered. There are two objections to the prone position; first, it is not well adapted for clearing cavities by expectoration, some of the sputum being liable to return to another bronchus, thus making a new focus of infection; second, in incipient disease it is impossible to maintain the muscles and the functions in proper order without exercise. Therefore, some physicians use this *Liegenhalle* system alone, while others add to it hill-climbing and other forms of pulmonary gymnastics. The exercises are regulated, the patient being enjoined to ascend slowly, not to talk while climbing, to breathe through the nose, and to stop short of actual fatigue. During steady walking five or six deep breaths are to be taken every 100 or 150 paces, breathing through the nose, or when lying in the open air taking from ten to twelve deep breaths every five or ten minutes.

The patient is stuffed with a rich and varied diet, and hydropathy is used, especially douches to the thorax to stimulate respiration. The floors of these sanitaria are covered with linoleum, the walls are painted with oil-colors, or are inlaid with wooden panels or papered with washable material; there is no sweeping done, but swabbing, instead, with damp cloths. The sputum is spat into vessels and afterward destroyed; it is frequently examined and when no bacilli can be found 1 cc. of the sputum is injected under the skin of a guinea-pig; and if in from three to six weeks the animal has not contracted tuberculosis the patient is considered fit to return home.—*Practical Medicine.*

